# Effects of the COVID-19 Lockdown on HbA1c Levels of Ethnic Minorities and Low-income Groups with Type 2 Diabetes in Israel

**DOI:** 10.1007/s40615-024-02238-z

**Published:** 2024-12-06

**Authors:** Galia Riklin, Michael Friger, Ilana Shoham-Vardi, Rachel Golan, Tamar Wainstock

**Affiliations:** 1 Department of Health Maintenance Organizations, Clalit Health Services, Dan Petah Tiqwa District, Ramat-Gan, Israel; 2 School of Public Health, Faculty of Health Sciences, Ben-Gurion University of the Negev, Beer-Sheva, Israel

**Keywords:** Lockdown, Type-2 Diabetes, Socioeconomic Status, Ethnic Minorities, Quantile Regression

## Abstract

**Aims:**

To investigate the impact of low socioeconomic status (SES) and/or membership in ethnic minority has on HbA1c before and during the COVID-19 lockdown.

**Methods:**

A retrospective cohort study was conducted between March 2019 and March 2021, based on data from electronic medical records of 17,072 patients with type-2 diabetes, collected by Clalit (Israel’s largest health maintenance organization). Low SES was compared to high and ethnic minorities (Arabs and ultra-Orthodox Jews) were compared to the general Israeli population of mostly Jewish, but not ultra-Orthodox, Israeli citizens. Quantile regressions were used to examine the impact of SES and ethnic minority membership on HbA1c levels in the 0.10, 0.25, 0.50, 0.75, 0.90 quantiles before and during the lockdown.

**Results:**

In the pre-lockdown period, patients with type-2 diabetes of low versus high SES, and Arabs versus the general population, had higher HbA1c. During the lockdown HbA1c levels of low versus high SES rose significantly in the 0.10 and 0.90 quantiles, and among Arabs HbA1c levels rose significantly across all quantiles, with a remarkable increment in the 0.90 quantile (from 0.316% in the pre-lockdown period to 0.730% in the lockdown period). Ultra-Orthodox Jewish diabetic patients had a marginally higher mean HbA1C level regardless of the period. Quantile regressions did not reveal a significant difference between the ultra-Orthodox Jewish and the general population.

**Conclusion:**

The lockdown exacerbated disparities in glycemic control between low and high SES individuals and between Arab minority and the general population.

**Supplementary Information:**

The online version contains supplementary material available at 10.1007/s40615-024-02238-z.

## Introduction

Type 2 diabetes is a metabolic disorder that manifests initially as elevated blood glucose levels. An estimated 536.6 million people were living with diabetes worldwide in 2021 [1], and almost 4.2 million adults died owing to this disease in 2019 [2]. Type 2 diabetes is a major cause of multiple concomitant illnesses [3–5] and is associated with increased mortality and deterioration in the quality of life. Nonmedical factors, such as income, standard of living, gender, and ethnicity, have a significant effect on the glycemic control of people with type 2 diabetes [6–8]. There are disparities in glycemic control between ethnic majorities and minorities, which are partially explained by the latter’s lower education levels, higher unemployment and poverty levels, different dietary norms, and limited access to healthcare service, compared with ethnic majorities [9]. Further, exposure to stressful situations, such as the national lockdowns because of the coronavirus disease (COVID-19) pandemic, may increase these gaps [10].

Nations worldwide imposed these lockdowns to contain the spread of the COVID-19 pandemic. In Israel, from March 2020 until December 2020, the government enacted emergency regulations. The lockdown policy drastically limited people’s freedom of movement and of gathering, and it included the closure of cultural, leisure, and entertainment venues, the cancellation of international flights, and the imposition of job market constraints. These national lockdowns were considered stressful and led to psychological distress due to the feeling of loneliness and financial instability [11].

In Israel, two main minority populations—Arabs and Ultra-Orthodox Jews—are potentially at greater risk for deteriorating glycemic control during the COVID-19 lockdown. The incidence of diabetes is higher among the ultra-Orthodox and Arab minorities than among the non-ultra-Orthodox Jewish population [12].

Arabs are considered the largest ethnic minority in Israel (about 21% of the general population) [13, 14]. The majority of the Arabs in Israel reside in the (predominantly rural) periphery. The (predominantly urban) center of Israel is home to 11% of Israeli Arabs [15]. In 2018, almost 45.3% of Arab families lived in poverty [14]. The percentage of Israeli Arabs with an academic degree is half that of the Jewish population [14, 15]. However, Arabs residing in the center of Israel experience better socioeconomic conditions compared to those in the periphery [15].

The ultra-Orthodox Jewish population represents the second-largest minority in Israel (about 13% of the general population) [13, 14]. The ultra-Orthodox Jewish population in Israel resides mostly in predominantly ultra-Orthodox Jewish neighborhoods in cities. Approximately 44% of the ultra-Orthodox Jews live in poverty, compared to 22% of non-ultra-Orthodox Jews in Israel. The employment rate among ultra-Orthodox Jewish men is 50.7%, and their level of formal education is lower than that of non-ultra-Orthodox Jewish men [13].

The non-ultra-Orthodox Jewish population (henceforth, the general population) represents the majority in Israel (about 66% of Israeli citizens) [13, 14].

While the lockdown period was critical in preventing the spread of the COVID-19 pandemic, its potential impact on HbA1c levels, among ethnic minorities, is still unclear. Previous studies have reported mixed results on the differences in HbA1c levels and glycemic control among ethnic minorities during the lockdown period [16, 17].

The purpose of the present study is to investigate the impact of the COVID-19 lockdown on changes in HbA1c levels of patients with diabetes, particularly among different socioeconomic groups and among different ethnic minorities in Israel, namely, the Arab and ultra-Orthodox Jewish populations.

## Methods

### Study Design

A retrospective cohort study was conducted, based on the medical records of patients with diabetes in one of the districts served by Clalit, between March 1, 2019 and March 1, 2021. Clalit is the largest health maintenance organization (HMO) in Israel, providing health coverage to more than half of the Israeli population. The studied district (Dan Petah Tiqwa) is located in the center of Israel and includes a diverse socioeconomic and ethnic population, including non-ultra-Orthodox Jewish, Arab, and ultra-Orthodox Jewish populations.

Patients were included in the study if they met all of the following criteria: were diagnosed with Type 2 diabetic, were aged 17 and over, were not hospitalized between March 1, 2019 and March 1, 2021, and had at least one HbA1c test during each study period (i.e., pre-lockdown and lockdown). In order to understand the effect of the COVID-19 lockdown without additional confounding stressors, patients were excluded from the study if they met one of the following criteria: were hospitalized during the lockdown—given that hospitalization is considered a stressful condition, it may affect glycemic control [18, 19]; were diagnosed with serious psychiatric disorders, such as schizophrenia, major depression, or manic depression, because such disorders increase the risk of being overweight and being unable to maintain a healthy lifestyle [20], with type 2 diabetes being a common comorbidity associated with excess weight [21]; had a mean HbA1c level lower than the pre-diabetes level (5.7) during each study period (*n* = 276). (Supplementary Fig. 1). The study was approved by Institutional Review Board of Meir Hospital (approval no. 0011–21-COM1).

### Data Collection

Data were collected from the electronic medical records in the Clalit Health Services database, including on participants’ age, sex, SES, and ethnicity. Further, data on their number of purchases per year of insulin and non-insulin diabetic medications from the pharmacy, and their blood tests results, were also collected. The number of purchases during the year ranged from 0 to 12.

### Variable Definitions

The lockdown period (March 1, 2020 to March 1, 2021) was the treatment period. The pre-lockdown period (March 1, 2019 to March 1, 2020) was the control period. To investigate whether changes in HbA1c levels were independent of a temporal trend, an additional comparison was conducted between the pre-lockdown period and the preceding year (March 1, 2018 to March 1, 2019). (Supplementary Fig. 2).

Sociodemographic characteristics were sex, age group (< 25, 25–49, 50–64, 65–84, 85 +), and socioeconomic status (SES). SES is based on residential location, according to the Socio-Economic Cluster of the Central Bureau of Statistics and socioeconomic index division [22], which characterizes neighborhoods in cities as either economically disadvantaged (low SES), middle-income (medium SES), or affluent (high SES). The socioeconomic index allows to classify the population according to SES and to compare the SES of the population living in different areas of Israel [22].

In addition, the participants were categorized by ethnicity into one majority group and two minority groups: the general population, ultra-Orthodox Jews, and Arabs, as determined by residential location [22]. According to the Central Bureau of Statistics, in the Arabs’ residential locations included in this study, 98–99% of the population are Arabs, whereas in the Jewish residential locations, less than 1% of the population are Arabs [23]. Further, according to the ultra-Orthodox websites, in the ultra-Orthodox areas included in this study, about 91% of the population are ultra-Orthodox, whereas in the general population areas, the proportion of the ultra-Orthodox population ranges from 0% to 8.7% [24].

Medications included insulin (A10A) and non-insulin diabetes medication (A10B), according to Anatomical Therapeutic Chemical (ATC) index codes. Medication use was defined as an ordinal variable the number of purchases of a monthly dose from the pharmacy as recorded by Clalit. The number of purchases per year ranged from 0 to 12. Those who did not buy insulin during the pre-lockdown period and bought it during the lockdown period were included in this study, given that paired regression analyses were conducted. The interaction between insulin and other diabetic medications was checked; however, since it did not improve the model goodness of fit, it was removed for simplicity.

Blood samples were collected and calculated as mean values for the pre-lockdown and lockdown study periods. The parameters included the mean glycated hemoglobin (HbA1c) value, the lipid profile as the mean triglycerides/high density lipoprotein (TGL/HDL) ratio (a high ratio is related to the severity of metabolic syndrome and insulin resistance [25]), and the mean blood albumin value. A low blood albumin level is considered as an important risk factor for severe diabetes-related complications in particular, diabetic nephropathy and diabetic foot syndrome [3].

### Statistical Analysis

Blood tests and age were represented as a mean ± standard deviation (SD). Ethnic groups and SES categories were represented as a number (*N*) and percentage of the study population.

A chi-square test was conducted to investigate independence within categorical variables (different SES categories and ethnic groups).

A one-way ANOVA test was used to compare the means of HbA1c levels between different SES categories and ethnic groups. Unpaired t-tests were used in a post-hoc analysis to compare low and medium SES, to high SES and the Arab and ultra-Orthodox Jewish to general population sectors.

A paired sample t-test was used to assess the differences in the mean HbA1c level between study periods.

A separate quantile regression for each study period was performed to examine the effect of SES and membership in an ethnic minority on HbA1c levels in the 0.10, 0.25, 0.50, 0.75, 0.90 quantiles before and during the lockdown. These quantile regression analyses were adjusted for sex, age, diabetic medication purchased (insulin or non-insulin), blood albumin, and TGL/HDL ratio. In the quantile regression, age was divided by cohort, < 25, 25–50, 50–65, 65–85, 85 + .

A one-way ANOVA between the quantiles, incorporating a Bonferroni correction, was conducted to determine the differences between quantiles.

To compare the impact of SES category and ethnic group on HbA1c levels between the two study periods, a comparative analysis of quantile regression coefficients was performed.

A change in the quantile regression coefficient was considered as significant if its value during the lockdown was outside the confidence interval of the pre-lockdown quantile regression coefficient value.

## Results

### Participant Characteristics

A comparison of HbA1c levels during the pre-lockdown and lockdown periods by SES category and by ethnic group is shown in Table 1.

**Table 1:** Characteristics and laboratory tests of people with type 2 diabetes during the pre-lockdown and lockdown periods by ethnic group and by SES category

Pre-lockdown period					Lockdown period			
Ethnic group								
	General^1^	Jewish Utra-Orthodox^1^	Arab^1^	*p*-value^2^	General^1^	Ultra-Orthodox Jewish^1^	Arab^1^	*p*-value^2^
Total Number	14 090 (82.6%)	1 804 (10.6%)	1 160 (6.8%)		14 090 (82.6%)	1 804 (10.6%)	1 160 (6.8%)	
SES				< 0·0001				< 0·0001
*High*	6 332 (45%)	0 (0%)	0 (0%)		6 332 (45%)	0 (0%)	0 (0%)	
*Medium*	5 843 (42%)	644 (36%)	1 160 (100%)		5 843 (42%)	644 (36%)	1 160 (100%)	
*Low*	1 866 (13%)	1 160 (64%)	0 (0%)		1 866 (13%)	1 160 (64%)	0 (0%)	
Sex				0·0014				0·0014
*Female*	7 064 (50%)	868 (48%)	636 (55%)		7 064 (50%)	868 (48%)	636 (55%)	
*Male*	7 026 (50%)	936 (52%)	524 (45%)		7 026 (50%)	936 (52%)	524 (45%)	
Age (years)	70 (12)	66 (12)	60 (12)	< 0·0001	71 (12)	67 (12)	61 (12)	< 0·0001
blood albumin	4·09 (0·31)	4·10 (0·30)	4·08 (0·31)	0·074	4·08 (0·35)	4·08 (0·34)	4·06 (0·33)	0·16
TGL/HDL ratio	3.10 (1.8)	2.97 (1.85)	3.56 (1.8)	< 0·0001	3.06 (1.79)	2.93 (1.83)	3.57 (1.81)	< 0·0001
Socioeconomic status								
	High^1^	Medium^1^	Low^1^	*p*-value^2^	High^1^	Medium^1^	Low^1^	p-value^2^
Total number	6 332 (37.2%)	7 647 (45.0%)	3 026 (17.8%)		6 332 (37.2%)	7 647 (45.0%)	3 026 (17.8%)	
Ethnic group				< 0·0001				< 0·0001
*General*	6 332 (100%)	5 843 (76%)	1 866 (62%)		6 332 (100%)	5 843 (76%)	1 866 (62%)	
*Ultra-Orthodox*	0 (0%)	644 (8·4%)	1 160 (38%)		0 (0%)	644 (8·4%)	1 160 (38%)	
*Arab*	0 (0%)	1 160 (15%)	0 (0%)		0 (0%)	1 160 (15%)	0 (0%)	
Sex				0·35				0·35
*Female*	3 148 (50%)	3 890 (51%)	1 509 (50%)		3 148 (50%)	3 890 (51%)	1 509 (50%)	
*Male*	3 184 (50%)	3 757 (49%)	1 517 (50%)		3 184 (50%)	3 757 (49%)	1 517 (50%)	
Age (years)	70 (12)	68 (12)	68 (11)	< 0·0001	71 (12)	69 (12)	69 (11)	< 0·0001
Blood albumin	4·10 (0·29)	4·08 (0·33)	4·09 (0·30)	0·0036	4·09 (0·33)	4·06 (0·36)	4·08 (0·34)	0·0002
TGL/HDL ratio	3.08 (1.8)	3.18 (1.8)	3.05(1.81)	0·0007	3.03 (1.81)	3.14 (1.80)	2.99 (1.79)	< 0·0001

^1^Mean (standard deviation); *n* (%)

^2^One-way ANOVA, Pearson’s chi-squared test

The sample size consisted of 17,072 patients with type-2 diabetes. Most of the participants were medium (45%) and high (37.5%) SES, while 17.8% were low SES. More than 82% of the patients with diabetes belonged to the general population, 10.6% were ultra-Orthodox Jewish, and 6.8% were Arab.

The entire Arab ethnic group in the present study was considered as medium SES. This classification was established based on the higher SES of Arabs living in central Israel in comparison to Israeli Arabs residing in peripheral areas [23]. The majority of ultra-Orthodox Jews (64%) were classified as low SES, and the remaining 36% as medium SES.

### A Comparison of Mean HbA1c Levels

In the pre-lockdown period, the mean HbA1c level among the Arabs (7.31%) and ultra-Orthodox Jews (7.13%) was higher than that of general population (7.04%) (*p* < 0.001 and *p* = 0.001, respectively). The mean HbA1c level of low-SES patients with diabetes (7.19%) was higher than the mean HbA1c level of high-SES patients with diabetes (6.95%) (*p* < 0.001).

In the lockdown period, the mean HbA1c level among Arabs (7.39%) and ultra-Orthodox Jews (7.06%) was higher than that of the general population (6.97%) (*p* < 0.001, *p* = 0.002, respectively). The mean HbA1c level among low-SES patients with diabetes (7.14%) was also higher than the mean HbA1c level among high-SES patients (6.87%) (*p* < 0.001).

**Fig. 1:**
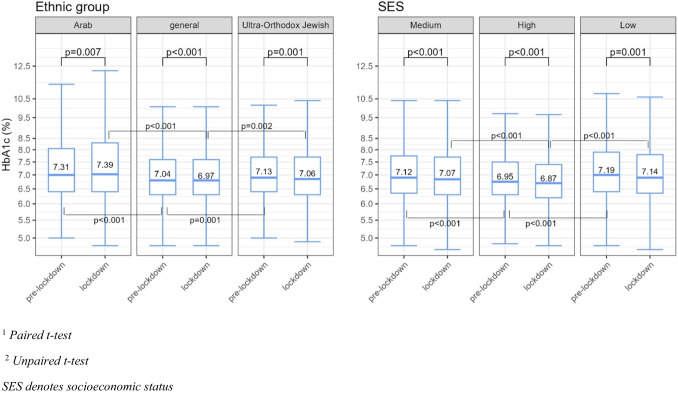
Comparison of mean HbA1c level of low and medium with high-SES patients with diabetes^2^; of Ultra-Orthodox Jewish and Arabs minorities with the general population^2^; in both pre-lockdown^1^ and lockdown periods^1^: boxplots; means; *p*-values^1,2^

In the lockdown period, a 0.07% decrease in mean HbA1c levels was observed in both the ultra-Orthodox Jewish and general populations (*p* = 0.001) and *p* < 0.001, respectively). Similar declines were observed among high- and low-SES patients (0.08% (*p* < 0.001) and 0.05% (*p* = 0.001), respectively). The only ethnic group exhibiting an increase in mean HbA1c levels during the lockdown was patients with diabetes from the Arab minority, showing a 0.08% increase (*p* = 0.007) (Fig. 1).

A comparison between the pre-lockdown period and the preceding year (March 1, 2018 to March 1, 2019) was performed to examine whether the changes in HbA1c levels were independent of a temporal trend. The mean HbA1c level remained stable or higher in the pre-lockdown period across all socioeconomic categories and ethnic groups (Supplementary Fig. 2).

### Analysis of Changes in HbA1c Levels Using a Multivariable Quantile Regression (QR)

The full results of the QR are shown in Table 3. The inter-quantile ANOVA test revealed significant differences between all the quantiles, in both the lockdown and the pre-lockdown periods (see Table 2).

**Table 2:** Mean HbA1c levels in the lockdown and pre-lockdown periods, standard deviations (*SD*), F-values, and p-values from an inter-quantile ANOVA test; pseudo *R*^2^ of each quantile

Quantiles	Lockdown period				Pre-lockdown period			
	Mean HbA1c level	SD	F^ab^	Pseudo R^2^	Mean HbA1c level	SD	F^ab^	Pseudo R^2^
*0.10*	5.333	1.16	14.007^***^	0.034	5.072	1.20	9.158^***^	0.035
*0.25*	6.069	1.14	23.651^***^	0.62	5.627	1.17	21.038^***^	0.059
*0.50*	7.008	1.13	27.065^***^	0.107	6.67	1.16	28.013^***^	0.099
*0.75*	8.501	1.14	16.705^***^	0.127	9.03	1.17	13.081^***^	0.126
*0.90*	11.447	1.19		0.115	11.42	1.24		0.129

^a^ANOVA test with Bonferroni correction

^b^Comparison between two models of consecutive quantiles

^***^ p < 0.001

**Fig. 2:**
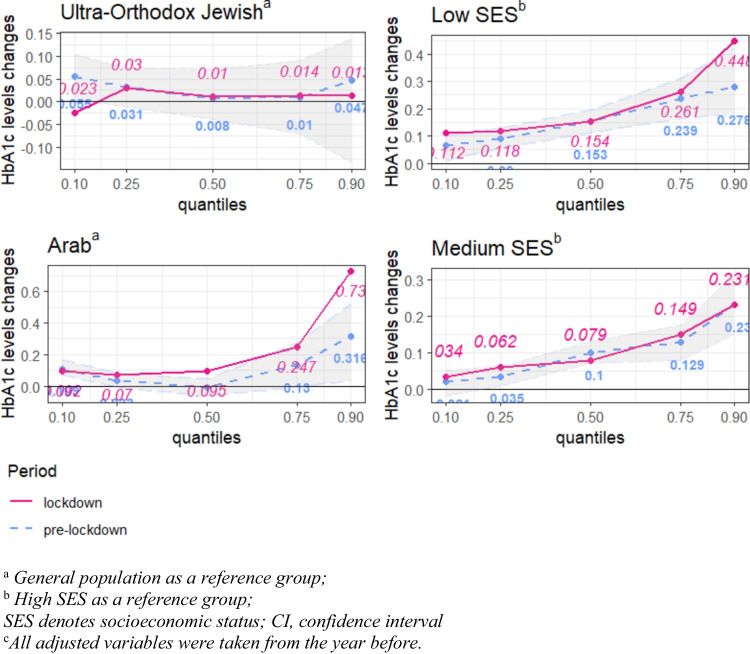
Quantile regression. Changes in HbA1c levels in the 0.10,0.25,0.50,0.75,0.90 quantiles, among patients with diabetes belonging to Arab and Ultra-Orthodox Jewish minorities^a^, low and medium SES^b^. Adjusted for sex, age, diabetic medication purchase (insulin and non-insulin), blood albumin, and TGL/HDL ratio^c^. Comparison between quantile regression coefficients in the pre-lockdown and lockdown periods. 95% CI of the pre- lockdown period. Significant changes between the periods are shown in bold. ^a^General population as a reference group

In the pre-lockdown period, low SES was significantly associated with higher HbA1c levels. During the lockdown this association became more pronounced, particularly among low-SES patients with diabetes in the 0.10 and 0.90 HbA1c quantiles. Specifically, the impact of low SES on the 10% of patients with diabetes with the highest HbA1c levels increased by more than 60% during the lockdown, with the quantile regression coefficient rising from 0.278 to 0.448 (Fig. 2, Table 3).

**Table 3:** Quantile regression. Changes in HbA1c levels in the 0.10, 0.25, 0.50, 0.75, 0.90 quantiles, adjusted for sex, age, diabetic medication purchase (insulin and non-insulin), blood albumin, and TGL/HDL ratio. Comparison between quantile regression coefficients in the pre-lockdown and lockdown periods. Significant differences between the periods are shown in bold

	HbA1c levels in the pre-lockdown period											HbA1c levels in the pre-lockdown period								
Quantiles	0.10		95%CI		0.25	95%CI		0.50		95%CI		0.10	95%CI		0.25	95%CI		0.50		95%CI
Intercept	5.333		4.402, 5.924		6.069	5.546, 6.377		7.008		6.289, 7.357		5.072	4.285, 5.508		5.627	5.407, 5.932		6.67		6.096, 7.601
Sex																				
Female	Reference group																			
Male	−0.013		−0.42, 0.063		0.059**	00.31, 0.087		−0.047*		−0.031, 0.087		−0.047*	−0.079, −0.021		−0.007	−0.033,0.019		0.015		−0.008, 0.04
**Age group**																				
> 25	Reference group																			
25–50	−0.108		−0.654, 0.688		−0.289	−0.515, 0.174		−0.429		−0.856, 0.197		−0.237	−0.588, .419		−0.02	−0.304, 0.152		−0.1		−0.998. 0.384
50–65	0.01		−0.538, 0.8		−0.191	−0.414, 0.27		−0.395		−0.82, 0.3227		−0.039	−0.394, 0602		0.148	−0.123, 0.294		−00.29		−0.904, 0.45
65–85	0.056		−0.488, 0.844		−0.21	−0.43, 0.25		−0.452		−0.804, 0.171		−0.019	−0.366, 0.621		0.111	−0.16, 0.256		−0.11		−0.985, 0.371
85 +	0.031		−0.514, 0.83		−0.23	−0.456, 0.237		−0.484		−0.913, 0.1391		−0.12	−0.474, 0.532		0.055	−0.218, 0.216		−0.127		−1.01, 0.356
SES																				
High	Reference group																			
Medium	0.021		−00.19, 0.054		0.035	0.008, 0.063		0.1**		0.071, 0.132		0.034	−0.002, 0.0066		0.062**	0.031, 0.088		0.079**		0.052, 0.106
Low	0.064*		0.009, 0.102		0.01**	0.054, 0.129		0.153**		0.109, 0.197		0.112**	0.006, 0.155		0.118**	0.068, 016		0.154**		0.0117, 0.195
**Cultural-ethnic groups**																				
general	Reference group																			
Ultra-orthodox	0.055		0.006, 0.105		0.031	−0.011, 0.073		0.008		−0.04, 0.07		−0.023	−0.63, 0.044		0.03	−0.018, 0.093		0.01		−0.037, 0.067
Arabs	0.109**		0.069, 0.168		0.033	−0.01, 0.083		−0.006		-		0.092*	0.004, 0.149		0.07	0.017, 0.147		0.095		0.028, 0.179
Diabetic medication																				
*Non-insulin*	0.02**		*0.017,0.024*		0.029**	*0.026, 0.031*		0.046**		*0.043,0.049*		0.019**	*0015, 0.022*		0.025**	*0.022, 0.028*		0.04**		***0.038, 0.043***
*Insulin*	0.056**		*0.052,0.066*		0.091**	*0.086, 0.096*		0.127**		*0.119, 0.134*		0.064**	*0.057, 0.069*		0.093**	*0.086, 0.099*		0.124**		***0.119, 0.131***
**Lab Tests**																				
*BA*	0.103**		*0.059, 0.146*		**0.042**	*−0.007, 0.073*		−0.073*		*−0.126, −0.019*		0.164**	*0.121, 0.223*		0.053*	*0.007, 0.097*		−0.086**		***−0.13, −0.043***
*TGL/HDL*	**0.006**		*−0.001, 0.013*		0.014**	*0.008,0.018*		0.03**		*0.022, 0.036*		**0.009**	*0.001, 0.016*		0.019**	*0.013, 0.026*		0.035**		***0.029, 0.043***
		HbA1c levels in the pre-lockdown period									HbA1c levels in the lockdown period									
***Quantiles***		***0.75***		***95%CI***			*0.90*		***95%CI***		***0.75***			***95%CI***			***0.90***		*95%CI*	
**Intercept**		8.501		7.862, 10.966			11.447		10.287, 13.69		9.03			7.641, 10.535			11.42		**10.255, 14.126**	
**Sex**																				
*Female*		*Reference group*																		
*Male*		0.113**		0.067, 0.157			0.232**		0.15, 0.302		0.072**			0.029, 0.105			0.165**		**0.088, 0.231**	
**Age Groups**		Reference group																		
< *25*																				
*25–50*		−0.395		−2.636, 0.067			−1.398		−3.469, 0.576		−1.09			−2.502, 0.356			−0.803		**−3.383, 0.286**	
*50–65*		−0.539**		−2.755, −0.091			−1.942		−4.015, 0.006		**−1.314**			−2.68, 0.12			−1.475*		**−4.061, −0.391**	
*65–85*		−0.738**		−2.881, −0.29			−2.326**		−4.383, −0.379		−1.486*			−2.848, −0.055			−1.886**		**−4.46, −0.797**	
*85* +		−0.775**		−3, −0.314			−2.421**		−4.484, −0.462		−1.515**			−2.876, −0.071			−1.89*		**−4.487, −0.774**	
**SES**																				
*High*		*Reference group*																		
*Medium*		0.129**		0.079, 0.175			0.231**		0.15, 0.316		0.149**			0.108, 0.188			0.231**		**0.147, 0.303**	
*Low*		0.239**		0.163, 0.311			0.278**		0.181, 0.425		0.261**			0.198, 0.323			0.448**		**0.318, 0.538**	
**Ethnic group**																				
*General*		*Reference group*																		
*Ultra-Orthodox*		0.01		−0.071, 0.091			0.047		−0.134, 0.139		0.014			−0.059, 0.083			0.013		**−0.091, 0.203**	
*Arab*		**0.13**		−0.009, 0.273			0.316*		0.033, 0.525		0.247**			0.152, 0.371			0.73**		**0.479, 0.992**	
**Diabetic medication**																				
*Non-insulin*		0.067**		0.062, 0.072			0.065**		0.055, 0.073		0.062**			0.058, 0.065			0.067**		**0.059, 0.076**	
*Insulin*		0.167**		0.154, 0.177			0.187**		0.163, 0.208		0.167**			0.156, 0.18			0.204**		**0.188, 0.224**	
**Lab Tests**																				
*BA*		−0.299**		−0.395, −0.226			−0.509**		−0.654, −0.402		−0.265**			−0.334, −0.184			−0.643**		**−0.729, −0.522**	
*TGL/HDL*		0.051**		0.037, 0.062			0.086**		0.067, 0.103		0.06**			0.046, 0.069			0.087**		**0.072, 0.112**	

*p < 0.05;**p < 0.01; HbA1c, glycated hemoglobin; pre-lockdown period, March 1, 2019 to March 1, 2020; lockdown period, March 1, 2020 to March 1, 2021; SES denotes socioeconomic status; BA, blood albumin; TGL/HDL, Triglycerides high-density lipoprotein cholesterol ratio; CI, confidence interval. All adjusted variables were taken from the year before

In the pre-lockdown period, Arab patients with diabetes exhibited significantly elevated HbA1c levels, both in the 0.10 and 0.90 quantiles, compared to the general population. During the lockdown, elevated HbA1c levels were observed and were significant in all quantiles. Particularly among patients with diabetes in the 0.90 quantile, the impact of belonging to the Arab minority on HbA1c levels was 2.3 times more pronounced in the lockdown compared to the pre-lockdown period, with the quantile regression coefficient rising from 0.316% to 0.730% (Fig. 2, Table 3).

No significant association was found between belonging to the ultra-Orthodox Jewish minority and HbA1c levels in either the pre-lockdown period or the lockdown period (Fig. 2, Table 3).

HbA1c levels were observed as positively associated with the purchase of diabetes medications for both insulin and non-insulin.

## Discussion

In this study, we observed an improvement in glycemic control in a part of the population during the COVID-19 lockdown. The HbA1c level decreased during the lockdown among patients with diabetes in the group aged 50–65 years with high HbA1c values (0.75 and 0.90 quantiles), as well as among men with diabetes with low HbA1c values (0.10–0.50 quantiles) (Table 3) The positive impact of the COVID-19 lockdown on the HbA1c l levels of patients with diabetes is in line with previous studies that also found a positive impact of the COVID**-**19 lockdown on glycemic control [26].

However, the findings on the effect of the lockdown on HbA1c levels are indicative of social inequality. Low-SES patients with diabetes whose HbA1c levels were in the highest 10% (they are represented in the 0.90 quantile of the quantile regression; see Fig. 2, Table 3) experienced a statistically significant 1.6-fold increase in their HbA1c levels during the lockdown compared with the corresponding levels in the pre-lockdown period.

Even in the pre-lockdown period, low-SES patients had higher HbA1c levels compared to high- and medium-SES patients with diabetes. This difference is despite the universal health care coverage provided to all Israeli citizens under its national health insurance law, which provides access to basic medical services regardless of the individual’s income [27].These findings indicate that low socioeconomic status can be an independent risk factor for the development of diabetes-related complications and mortality [7, 8].

Moreover, prolonged exposure to atypical, stressful conditions, such as during the lockdown, which limited people’s opportunities to engage in physical and social activities, combined with economic hardship, may have adverse effects on the long-term health management of patients with diabetes, including on glycemic control.

During the COVID-19 pandemic, medical services focused most of their efforts on reducing the infection rate, and health promotion initiatives received less emphasis [28]. In the present study, the significant deterioration in glycemic control during the lockdown was mainly observed among low-SES patients with diabetes whose HbA1c levels were in the highest 10%. For low-SES patients with diabetes with high HbA1c levels, participation in physical activity, adherence to a healthy diet, and timely medication intake have been shown to be suboptimal prior to the stressful lockdown period [29].

While the National Health Insurance law provides access to basic medical services and medications to all Israeli citizens, [27], a copayment applies; further, some diabetes medications are only partly covered, which means that patients must pay the remaining amount, which might make these medications unaffordable for financially insecure people. In Israel, in 2019, the annual healthcare expenditure was 3,378 USD per capita, of which the government covered approximately 2,791 USD, leaving 587 USD to be paid out of pocket by the citizen [30].

In addition, low SES is often associated with limited knowledge about diabetes, which is associated with suboptimal self-care behaviors, such as physical inactivity and poor adherence to a healthy diet, which, in turn, result in poor glycemic control [8, 31].

Furthermore, this study revealed that as HbA1c levels increased, patients purchased more insulin and non-insulin diabetic medications. Thus, it can be assumed that as their HbA1c levels increased, the more likely patients were to attempt managing the diabetes through pharmacological treatment. However, non-medical factors, such as maintaining a healthy lifestyle, participating in physical activity, and adhering to a healthy diet, also play a significant role in diabetes management [4, 7, 31].

For these reasons, health promotion and education, ensuring the availability of, and accessibility to, facilities for physical activity and improving access to health care are vital interventions for this specific group of patients with diabetes during both normal and stressful periods.

In addition, the Arab minority patients had higher HbA1c levels than the general population and the ultra-Orthodox Jewish minority across all periods. During the pre-lockdown period, Arab patients with type 2 diabetes and the 10% lowest or the 10% highest HbA1c levels had higher HbA1c levels than the general population. In the lockdown period, their HbA1c levels increased even more and became significant among Arab patients with diabetes across all quantiles, ranging from 0.095 in the 0.50 quantile to almost 0.250 in the 0.75 quantile and increasing from 0.316 in the pre-lockdown period to 0.730 in the lockdown period in the 0.90 quantile. The increments in the HbA1c levels by 0.250 and 0.730 enhance the risk of diabetes-related complications and mortality [32, 33], cardiovascular disease incidence [33], microvascular limb injury [34], development of microalbuminuria and the progression of renal disease [35].

In line with previous studies (e.g. [15, 36],) we find evidence of poorer glycemic control among Israeli Arabs compared to Jews in Israel.

There are several possible explanations for these disparities in glycemic control and diabetes-related complications and mortality rates between the Arab minority and the general population in the pre-lockdown period, and the exacerbation of these disparities in the lockdown period. One explanation is that similar disparities have been observed in other ethnic minorities [6]. Specifically, the Arab minority is characterized by lower SES compared to the general population, and is often employed in temporary or unregulated positions, which were more severely impacted economically during the COVID-19 pandemic in Israel [15]. Additionally, the deterioration of the economic situation may have induced feelings of stress, which could potentially exacerbate the individual's health condition [37]. Another potential explanation for the higher HbA1c levels among Arab Israelis is the fact that Arab cuisine is based on traditional calorie-dense foods [38, 39]. Moreover, the Arab cultural practice of honoring guests requires the host to offer guests meals and desserts during social gatherings [38, 40]. Likewise, the practice of showing respect to the host, requires the guests to eat the meals and desserts offered by the host [40], individuals with diabetes often choose to eat these meals that are high in sugar rather than offend their host [41].

Although social gatherings were restricted during the lockdown, Israeli Arabs were less adherent to social isolation due to discontent with the government, coupled with cultural norms [42]. However, further research is needed to confirm these hypothesizes.

A marginally higher mean HbA1c level was observed among the ultra-Orthodox Jewish minority compared to the general population. Similarly, in quantile regressions, no significant difference in HbA1c levels was found between patients with diabetes from the ultra-Orthodox Jewish minority and the general population. These findings are similar to previous studies [12, 43], which affirm a higher incidence of diabetes among the ultra-Orthodox Jewish minority than among the general population. However, this incidence is still lower than expected given the lower educational and socioeconomic levels within the ultra-Orthodox Jewish community [12]. The relatively low incidence of diabetes among the ultra-Orthodox minority has been explained by the meditative and calming effect of prayer [44, 45]. However, Chernichovsky and Sharony [46], hypothesize that ultra-Orthodox Jews are in relatively good health as a result of the significant social capital prevalent among this population. In particular, the ultra-Orthodox community in Israel is characterized by strong familial connections, and high involvement in the ultra-Orthodox community life, particularly through volunteering. It has been argued that this psychosocial support network fosters a sense of security and reduces psychological distress [46]. Further research is needed to confirm these hypotheses as well.

Notably, this study has several limitations, which mostly relate to our use of data from medical records. First, data on ethnicity and SES were not available in the medical records and had to be determined from the place of residence. We could determine ethnicity from the residential location because, in Israel, according to the ultra-Orthodox websites [24], almost the entire ultra-Orthodox Jewish population resides in ultra-Orthodox neighborhoods (about 91%), whereas according to the Central Bureau of Statistics, almost the entire Israeli Arab population (about 99%) resides in Arab neighborhoods [11, 14, 23]. The general population and the ultra-Orthodox population have a greater likelihood of overlapping, which can be considered a limitation for there may be “no difference” between the ultra-Orthodox population and the general majority population. This division of the population by residential area is the only classification available for use when conducting a mega-data analysis using pre-existing data files of Clalit HMO. According to the Israeli law all HMOs are obligated to accept patients regardless of age, gender, SES, sector, or religious observance, therefore there is no question about race ethnicity or SES in the medical record. Thus, geographic division is the only metric available for categorizing ethnicity.

Turning from ethnicity to socioeconomic status, the entire Arab ethnic group in the present study was categorized as medium SES. This categorization is based on the higher SES of Arabs residing in the center of Israel compared to those living in peripheral areas [14] and on the country’s socioeconomic index. In Israel, the population is classified into socioeconomic clusters based on a socioeconomic index. This index allows for comparison between people living in different geographic units [22]. The index serves as a reasonable proxy for the relative income and well-being of communities, as it heavily incorporates factors such as income, education, and unemployment [47]. The ultra-Orthodox Jewish ethnic group ranges between medium and low SES [15]. Belonging to an ethnic minority and low SES often overlap, making it challenging to distinguish between them [48, 49]. However, the unique demographics of the Israeli population allows for such differentiation. A second limitation is the low numbers of ultra-Orthodox Jewish and Arab participants compared to the general population.

Another limitation of this study is that weight and body mass index could not be included in the analysis due to the high percentage of missing values. Instead of weight, the TGL/HDL ratio was used. This rate is sensitive to weight changes and can be considered as an indicator of metabolic syndrome [25]. Data on medication usage was not available in the medical records; however, we were able to proxy for this using medication purchases from pharmacies.

Finally, this study represents only participants located in the center of Israel who underwent at least one HbA1c test in each study period and were not hospitalized. Therefore, the study population may not be fully representative and could possibly be biased toward patients with better glycemic control.

Despite the limitations, the large sample size and cohort design could provide insights for future studies and intervention planning. Future research should focus on identifying the long-term outcomes of the COVID-19 lockdown, and the behavioral changes in people with diabetes from ethnic minorities during such stressful situations. Moreover, valuable insights may be obtained from interventional studies among ethnic minority populations, which assess the impact of proactive education regarding optimal adjustments for maintaining a healthy lifestyle at times of limited access to health care. Further qualitative studies aiming to understand in depth the impact of the COVID-19 lockdown on diabetic management would also be beneficial to better understand the "why" questions.

In conclusion, the present study found that, overall, the condition of patients with diabetes improved during the lockdown for a large part of the Israeli population. However, among several subgroups the condition of patients with diabetes worsened during the lockdown, namely, the Arab minority group and low-SES with a history of poor glycemic control before the lockdown period. In the future, knowledge about the condition of patients with diabetes during a crisis can be used to provide targeted interventions, especially among vulnerable populations.

## Supplementary information

Below is the link to the electronic supplementary material.

Supplementary file1 (PDF 224 KB)

Supplementary file2 (PDF 432 KB)

## References

[1] Ogurtsova K, da Rocha Fernandes JD, Huang Y, Linnenkamp U, Guariguata L, Cho NH, ... Makaroff LE. IDF Diabetes Atlas: Global estimates for the prevalence of diabetes for 2015 and 2040. Diab Res Clinic Pract. 2017;128:40–50.‏ 10.1016/j.diabres.2017.03.024 10.1016/j.diabres.2017.03.024 28437734

[2] Saeedi P, Salpea P, Karuranga S, Petersohn I, Malanda B, Gregg EW, Unwin N, Wild SH, Williams R. Mortality attributable to diabetes in 20–79 years old adults, 2019 estimates: Results from the International Diabetes Federation Diabetes Atlas, 9th edition. Diabetes Res Clin Pract. 2020;162:108086. 10.1016/j.diabres.2020.108086 10.1016/j.diabres.2020.108086 32068099

[3] Xu S, Wang Y, Hu Z, Ma L, Zhang F, Liu P. Effects of neutrophil-to-lymphocyte ratio, serum calcium, and serum albumin on prognosis in patients with diabetic foot. Int Wound J. 2023;20(5):1638–46. 10.1111/iwj.14019. 36366862 10.1111/iwj.14019 PMC10088829

[4] Thomas MC, Brownlee M, Susztak K, Sharma K, Jandeleit-Dahm KAM, Zoungas S, Rossing P, Groop PH, Cooper ME. Diabetic kidney disease. Nat Rev Dis Primers. 2015;1:15018. 10.1038/nrdp.2015.18. 27188921 10.1038/nrdp.2015.18 PMC7724636

[5] Li S, Wang J, Zhang B, Li X, Liu Y. Diabetes mellitus and cause-specific mortality: A population-based study. Diabetes Metab J. 2019;43(3):319–41. 10.4093/dmj.2018.0060. 31210036 10.4093/dmj.2018.0060 PMC6581547

[6] Barber S, Roux AVD, Cardoso L, Santos S, Toste V, James S, Barreto S, Schmidt M, Giatti L, Chor D. At the intersection of place, race, and health in Brazil: Residential segregation and cardio-metabolic risk factors in the Brazilian Longitudinal Study of Adult Health (ELSA-Brasil). Soc Sci Med. 2018;199:67–76. 10.1016/j.socscimed.2017.05.047. 28821371 10.1016/j.socscimed.2017.05.047

[7] Kavanagh A, Bentley RJ, Turrell G, Shaw J, Dunstan D, Subramanian SV. Socioeconomic position, gender, health behaviours and biomarkers of cardiovascular disease and diabetes. Soc Sci Med. 2010;71(6):1150–60. 10.1016/j.socscimed.2010.05.038. 20667641 10.1016/j.socscimed.2010.05.038

[8] Sims M, Roux AVD, Boykin S, Sarpong D, Gebreab SY, Wyatt SB, Hickson DM, Payton M, Ekunwe L, Taylor HA. The socioeconomic gradient of diabetes prevalence, awareness, treatment, and control among African Americans in the Jackson heart study. Ann Epidemiol. 2011;21(12):892–8. 10.1016/j.annepidem.2011.05.006. 21737303 10.1016/j.annepidem.2011.05.006 PMC3192269

[9] Campbell JA, Yan A, Walker RE, Weinhardt L, Wang Y, Walker RJ, Egede LE. Relative contribution of individual, community, and health system factors on glycemic control among inner-city African Americans with type 2 diabetes. J Racial Ethn Health Disparities. 2021;8:402–14. 10.1007/s40615-020-00795-7. 32588396 10.1007/s40615-020-00795-7 PMC7759592

[10] Vajravelu ME, Mani I, Malik S, Hewitt B, Peyyety V, Arslanian S. Race-and neighborhood-related disparities spanning the COVID-19 pandemic: trajectories of combined glycemic Control and BMI in youth with diabetes. Diabetes Care. 2023;46(3):511–8. 10.2337/dc22-1439. 36534444 10.2337/dc22-1439 PMC10020019

[11] Dubey S, Biswas P, Ghosh R, Chatterjee S, Dubey MJ, Chatterjee S, Lahiri D, Lavie CJ. Psychosocial impact of COVID-19. Diabetes Metab Syndr. 2020;14(5):779–88. 10.1016/j.dsx.2020.05.03. 32526627 10.1016/j.dsx.2020.05.035 PMC7255207

[12] Peleg-Gabai M. Data on Diabetes in Israel. 2023. Accessed 5 Nov 2024. https://main.knesset.gov.il/EN/activity/mmm/DataonDiabetesinIsrael.pdf

[13] Cahaner L, Malach G. The Yearbook of Ultra-Orthodox Society in Israel 2024. 2022. Accessed 5 Nov 2024. https://en.idi.org.il/publications/38701

[14] Haj-Yahya NNH, Khalaily M, Rudnitzky A, Fargeon B. Statistical Report on Arab Society in Israel 2021. Israel Democracy Institute and the Authority for the Economic Development of the Minorities Sector, Ministry of Social Equality. 2022. Accessed 5 Nov 2024. https://en.idi.org.il/articles/38540

[15] Chernichovsky D, Bisharat B, Bowers L, Brill A, Sharony C. The health of the Arab Israeli population. State Nation Rep. 2017;325:8–20.

[16] Ledford CJW, Roberts C, Whisenant E, Walters C, Akamiro K, Butler J, Ali A, Seehusen DA. Quantifying worsened glycemic control during the COVID-19 pandemic. J Am Board Fam Med. 2021;34:S192–5. 10.3122/JABFM.2021.S1.200446. 33622837 10.3122/jabfm.2021.S1.200446

[17] Williams MS, Cigaran E, Martinez S, Marino J, Barbero P, Myers AK, DiClemente RJ, Goris N, Gomez VC, Granville D, Guzman J, Harris YT, Kline M, Lesser ML, Makaryus AN, Murray LM, McFarlane SI, Patel VH, Polo J, …& Pekmezaris, R. COVID-19 stressors for Hispanic/Latino patients living with type 2 diabetes: A qualitative study. Front Clinic Diab Healthcare. 2023;4:1070547. 10.3389/fcdhc.2023.1070547. 10.3389/fcdhc.2023.1070547 PMC10175775 37187937

[18] Mirani SH, Areja D, Gilani SS, Tahir A, Pathan M, Bhatti S. Frequency of depression and anxiety symptoms in surgical hospitalized patients. Cureus. 2019;11(2):e4141. 10.7759/cureus.4141. 31058024 10.7759/cureus.4141 PMC6485537

[19] Ingrosso DMF, Primavera M, Samvelyan S, Tagi VM, Chiarelli F. Stress and diabetes mellitus: Pathogenetic mechanisms and clinical outcome. Hormone Res Paediatr. 2023;96(1):34–43. 10.1159/000522431. 10.1159/000522431 35124671

[20] Bradshaw T, Mairs H. Obesity and serious mental ill health: A critical review of the literature. In Healthcare (Switzerland) 2014;2(2):166–182. MDPI. 10.3390/healthcare2020166 10.3390/healthcare2020166 PMC4934464 27429268

[21] Ruze R, Liu T, Zou X, Song J, Chen Y, Xu R, ... Xu Q. Obesity and type 2 diabetes mellitus: connections in epidemiology, pathogenesis, and treatments. Front Endocrinol. 2023;14:1161521.‏ 10.3389/fendo.2023.1161521 10.3389/fendo.2023.1161521 PMC10161731 37152942

[22] Tsibel N. Characterization and Classification of Geographical Units by the Socio-Economic Level of the Population 2021. Central Bureau of Statistics. 2024. Accessed 5 Nov 2024. https://www.cbs.gov.il/en/mediarelease/Pages/2024/Characterization-and-Classification-of-Geographical-Units-by-the-Socio-Economic-Level-of-the-Population-2021.aspx

[23] Regional Statistics. Central Bureau of Statistics. (Hebrew). 2022. Accessed 5 Nov 2024. https://www.cbs.gov.il/he/settlements/Pages/default.aspx?subject=%D7%9E%D7%93%D7%93%20%D7%97%D7%91%D7%A8%D7%AA%D7%99%20%D7%9B%D7%9C%D7%9B%D7%9C%D7%99

[24] Population Indicators by Sector and Municipality, 2021 (2023) Wohl Data Center. https://data.machon.org.il/en/dashbords/demographics-en/. Accessed 5 Nov 2024.

[25] Azarpazhooh MR, Najafi F, Darbandi M, Kiarasi S, Oduyemi T, Spence JD. Triglyceride/high-density lipoprotein cholesterol ratio: A clue to metabolic syndrome, insulin resistance, and severe atherosclerosis. Lipids. 2021;56(4):405–12. 10.1002/lipd.12302. 33881177 10.1002/lipd.12302

[26] Palanca A, Quinones-Torrelo C, Girbés J, Real JT, Ampudia-Blasco FJ. Impact of COVID-19 lockdown on diabetes management and follow-up in a broad population in Spain. Eur J Clin Invest. 2022;52(6):e13771. 10.1111/eci.13771. 35313009 10.1111/eci.13771 PMC9111861

[27] Sabbah MY. Health care system structure in the State of Israel. J Med Sci. 2019;88(1):39–46. 10.20883/jms.332

[28] Levin-Zamir D, Sorensen K, Su TT, Sentell T, Rowlands G, Messer M, Pleasant A, Nunes LS, Lev-Ari S, Okan O (2021). Health promotion preparedness for health crises: A “must” or “nice to have”? Case studies and global lessons learned from the COVID-19 pandemic. Glob Health Promot. 2021;28(2):27–37. https://journals.sagepub.com/doi/full/10.1177/1757975921998639 10.1177/1757975921998639 PMC8246413 33775167

[29] Rahman M, Nakamura K, Hasan SM, Seino K, Mostofa G. Mediators of the association between low socioeconomic status and poor glycemic control among type 2 diabetics in Bangladesh. Scientif Rep. 2020;10(1):6690.‏ 10.1038/s41598-020-63253-8 10.1038/s41598-020-63253-8 PMC7174358 32317650

[30] World Health Organization. Health systems in action: Israel. 2022 edition. Accessed 5 Nov 2024. https://iris.who.int/bitstream/handle/10665/362342/9789289059138-eng.pdf?sequence=1

[31] Silva-Tinoco R, Cuatecontzi-Xochitiotzi T, De la Torre-Saldaña V, León-García E, Serna-Alvarado J, Orea-Tejeda A, ... Prada D. Influence of social determinants, diabetes knowledge, health behaviors, and glycemic control in type 2 diabetes: an analysis from real-world evidence. BMC Endocr Disord. 2020;20:1–11. ‏10.1186/s12902-020-00604-6 10.1186/s12902-020-00604-6 PMC7449009 32843004

[32] Critchley JA, Carey IM, Harris T, DeWilde S, Cook DG. Variability in glycated hemoglobin and risk of poor outcomes among people with type 2 diabetes in a large primary care cohort study. Diabetes Care. 2019;42(12):2237–46. 10.2337/dc19-0848. 31582426 10.2337/dc19-0848

[33] Wan EYF, Yu EYT, Chin WY, NGFTY, Chia SMC, Wong I, ... Lam CLK (2020) Age-specific associations of HbA1c variability with cardiovascular disease and mortality in type 2 diabetes mellitus patients: a 10-year cohort study. Diabetes, Obes Metabol. 2020;22(8):1316–1327.‏ 10.1111/dom.14034 10.1111/dom.14034 32196917

[34] Dhatariya K, Humberstone A, Hasnat A, Wright R, Lujan M, Nunney I. The Association Between Mean Glycated Haemoglobin or Glycaemic Variability and the Development of Retinopathy in People with Diabetes: A Retrospective Observational Cohort Study. Diabetes Therapy. 2021;12:2755–66. 10.1007/s13300-021-01146-3. 34491530 10.1007/s13300-021-01146-3 PMC8479058

[35] Cheng D, Fei Y, Liu Y, Li J, Xue Q, Wang X, Wang N. HbA1C variability and the risk of renal status progression in diabetes mellitus: a meta-analysis. PLoS ONE. 2014;9(12):e115509. 10.1371/journal.pone.0115509. 25521346 10.1371/journal.pone.0115509 PMC4270779

[36] Jaffe A, Giveon S, Wulffhart L, Oberman B, Baidousi M, Ziv A, Kalter-Leibovici O. Adult Arabs have higher risk for diabetes mellitus than Jews in Israel. PLoS ONE. 2017;12(5):e0176661. 10.1371/journal.pone.0176661. 28481942 10.1371/journal.pone.0176661 PMC5421762

[37] Robinson DJ, Coons M, Haensel H, Vallis M, Yale JF. Diabetes and mental health. Can J Diabetes. 2018;42:S130–41. 10.1016/j.jcjd.2017.10.031. 29650085 10.1016/j.jcjd.2017.10.031

[38] Bays HE, Antoun J, Censani M, Bailony R, Alexander L. Obesity pillars roundtable: Obesity and individuals from the Mediterranean region and Middle East. Obesity Pillars. 2022;2:100013. 10.1016/j.obpill.2022.100013. 37990716 10.1016/j.obpill.2022.100013 PMC10661985

[39] Hoteit M, Zoghbi E, Rady A, Shankiti I, Al-Jawaldeh A. Fatty acids quality in Middle Eastern traditional dishes, Arabic sweets and market foods frequently consumed in Lebanon. Nutrients. 2021;13(7):2462. 10.3390/nu13072462. 34371969 10.3390/nu13072462 PMC8308895

[40] Sobh R, Belk RW, Wilson JAJ. Islamic Arab hospitality and multiculturalism. Mark Theory. 2013;13(4):443–63. 10.1177/1470593113499695.

[41] Khalil AB, Beshyah SA, Abdella N, Afandi B, Al-Arouj MM, Al-Awadi F, Benbarka M, ben Nakhi A, Fiad TM, al Futaisi A, Hassoun AA, Hussein W, Kaddaha G, Ksseiry I, Lamki M, Madanial AA, Saber F, Aal ZA, Morcos B, Saadi H. Diabesity in the Arabian Gulf: Challenges and opportunities. Oman Med J. 2018;33(4):273–282. 10.5001/omj.2018.5310.5001/omj.2018.53 10.5001/omj.2018.53 PMC6047189 30038726

[42] Lavie E, Elran M, Sawaed K, Even S. The Resiliency of the Arab Society in Israel during the Covid-19 pandemic. Institute for National Security Studies. (Hebrew). 2021. Accessed 5 Nov 2024. מזכר-208-חוסנה-של-החברה-הערבית-בישראל-במשבר-הקורונה.pdf (inss.org.il)

[43] Eilat-Adar S, Hellerstein D, Goldbourt U. Religiosity is associated with reduced risk of all-cause and coronary heart disease mortality among Jewish men. Int J Environ Res Public Health. 2022;19(19):12607. 10.3390/ijerph191912607. 36231908 10.3390/ijerph191912607 PMC9566524

[44] Darvyri P, Christodoulakis S, Galanakis M, Avgoustidis AG, Thanopoulou A, Chrousos GP. On the role of spirituality and religiosity in type 2 diabetes mellitus management: A systematic review. Psychology. 2018;09(04):728–44. 10.4236/psych.2018.94046.

[45] Mishra SK, Togneri E, Tripathi B, Trikamji B. Spirituality and religiosity and its role in health and diseases. J Relig Health 2017;56(4):1282–1301. 10.1007/s10943-015-0100-z 10.1007/s10943-015-0100-z 26345679

[46] Chernichovsky D, Sharony C. The Relationship between social capital and health in the Haredi sector. In Chernichovsky Dov & Weiss Avi (Eds.), State of the Nation Report Society, Economy and Policy in 2015 (pp. 435–465). Taub Center for Social Policy Studies in Israel. 2015. Accessed 5 Nov 2024. https://www.taubcenter.org.il/wp-content/uploads/2020/12/therelationshipbetweensocialcapitalandhealthintheharedisectorenglish.pdf

[47] Chernichovsky D, Anson J. The Jewish-Arab divide in life expectancy in Israel. Econ Hum Biol. 2005;3(1):123–37. 10.1016/j.ehb.2005.01.002. 15722265 10.1016/j.ehb.2005.01.002

[48] Braveman PA, Cubbin C, Egerter S, Williams DR, Pamuk E. Socioeconomic disparities in health in the United States: What the patterns tell us. Am J Publ Health. 2010;100:(Suppl. 1). 10.2105/AJPH.2009.166082 10.2105/AJPH.2009.166082 PMC2837459 20147693

[49] Fryer RG, Jr. Racial inequality in the 21st century: The declining significance of discrimination. In: Handbook of Labor Economics; 2011 (B, Vol. 4, pp. 855–971). 10.1016/S0169-7218(11)02408-7

