# Supplementary figures and images for: Effects of the COVID-19 Lockdown on HbA1c Levels of Ethnic Minorities and Low-income Groups with Type 2 Diabetes in Israel
Source: J Racial Ethn Health Disparities. 2024 Dec 6;13(1):218–29. doi: 10.1007/s40615-024-02238-z (PMC12795947; doi:10.1007/s40615-024-02238-z)

**Supplementary Fig1. The flow chart of the study design**

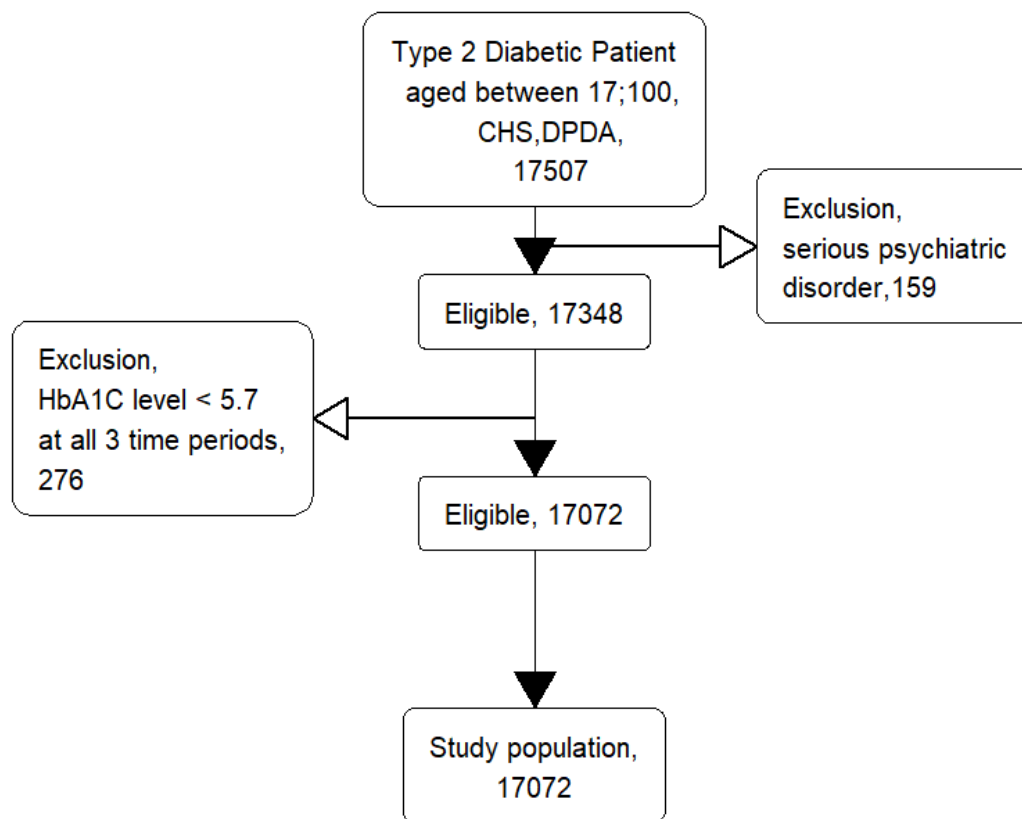

Supplement: Supplementary file 1 — Supplementary file1 (PDF 224 KB) [file 40615_2024_2238_MOESM1_ESM.pdf]
