# Supplementary material for: Effects of the COVID-19 Lockdown on HbA1c Levels of Ethnic Minorities and Low-income Groups with Type 2 Diabetes in Israel
Source: J Racial Ethn Health Disparities. 2024 Dec 6;13(1):218–29. doi: 10.1007/s40615-024-02238-z (PMC12795947; doi:10.1007/s40615-024-02238-z)
Supplement: Supplementary file 2 — Supplementary file2 (PDF 432 KB) [file 40615_2024_2238_MOESM2_ESM.pdf]

Supplementary Fig2. Changes in mean HbA1C across pre-pre-lockdown[3], pre-lockdown[2] and lockdown[1] periods, by sector and socioeconomic status: boxplots; means; p-values [a]

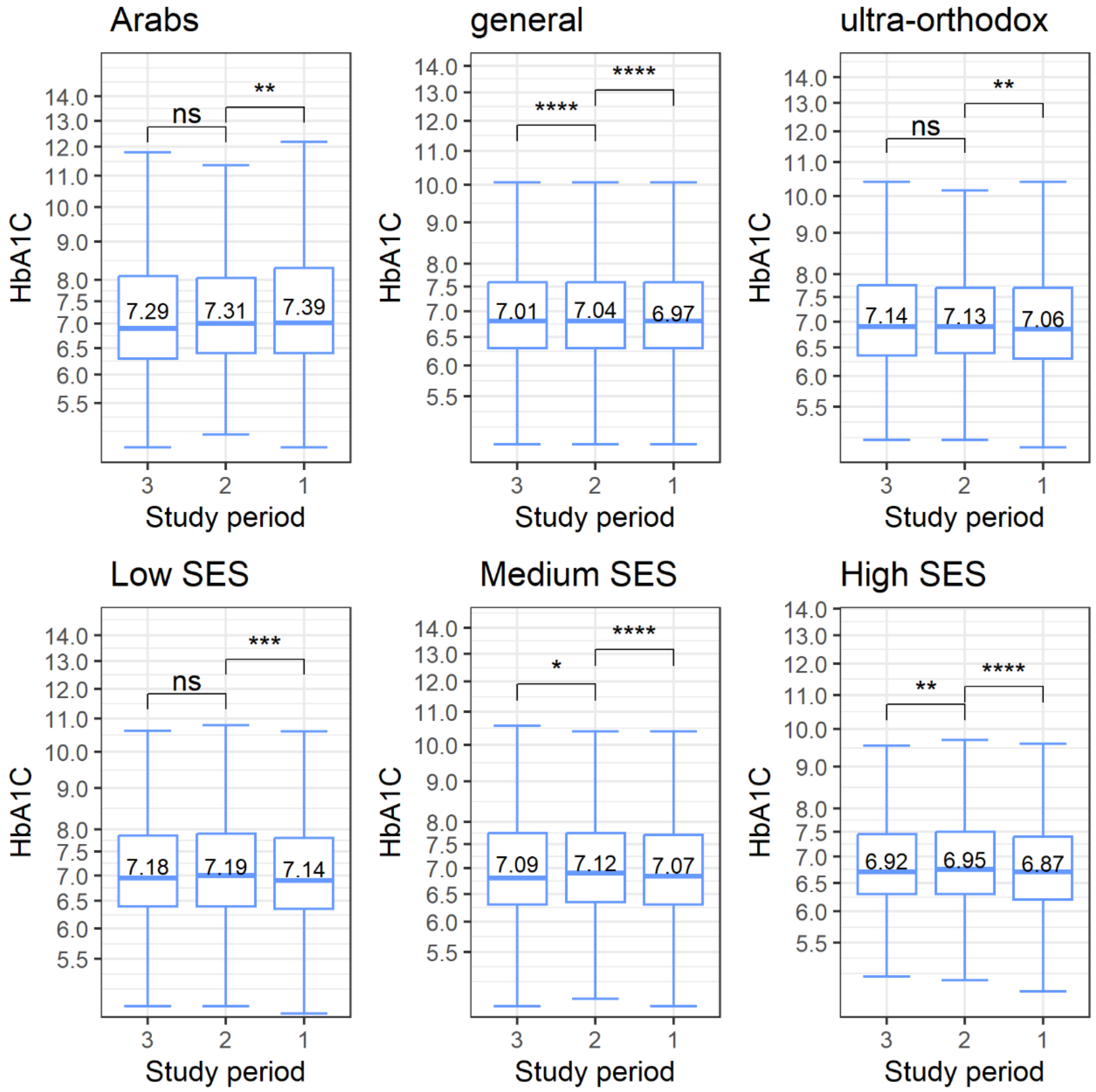

3, March 1st, 2018 to March 1st, 2019; 2 ,March 1st, 2019 to March 1st, 1; 2020, March 1st, 2020 to March 1st, 2021;
HbA1C, glycated hemoglobin. SES, Socioeconomic Status
[a]paired t-test , Bonferroni correction
ns, not significant p<0.0.5, **p<0.01, ***p<0.0001,*
